# Disclosure to genetic relatives without consent – Australian genetic professionals’ awareness of the health privacy law

**DOI:** 10.1186/s12910-020-0451-1

**Published:** 2020-02-04

**Authors:** Natalia Meggiolaro, Kristine Barlow-Stewart, Kate Dunlop, Ainsley J. Newson, Jane Fleming

**Affiliations:** 1 The University of Sydney, Northern Clinical School, Faculty of Medicine and Health, Level 7, Kolling Institute of Medical Research, Royal North Shore Hospital, Sydney, NSW Australia; 2 Centre for Genetics Education, NSW Health, Sydney, NSW Australia; 3 The University of Sydney, Faculty of Medicine and Health, Sydney School of Public Health, Sydney Health Ethics, Sydney, NSW Australia

**Keywords:** Privacy, Genetic information, Disclosure without consent, Genetic counseling, Duty to warn, Genetic testing, Confidentiality

## Abstract

**Background:**

When a genetic mutation is identified in a family member (proband), internationally, it is usually the proband’s or another responsible family member’s role to disclose the information to at-risk relatives. However, both active and passive non-disclosure in families occurs: choosing not to communicate the information or failing to communicate the information despite intention to do so, respectively. The ethical obligations to prevent harm to at-risk relatives and promote the duty of care by genetic health professionals (GHPs) is in conflict with Privacy laws and professional regulations that prohibits disclosure of information to a third party without the consent of the proband (duty of confidentiality). In New South Wales (NSW), Australia, amendments to Privacy legislation permits such disclosure to living genetic relatives with the process defined under guidelines although there is no legal duty to warn. This study assessed NSW GHP’s awareness and experience of the legislation and guidelines.

**Methods:**

An online survey collected demographics; theoretical knowledge; clinical scenarios to assess application knowledge; attitudes; confidence; experience with active non-disclosure. A link to correct answers was provided after completion. Knowledge scores above the median for non-parametric data or above the mean for parametric data were classified as ‘good’ or ‘poor’. Chi square tests assessed associations between confidence and knowledge scores.

**Results:**

While many of the 37 participants reported reading the guidelines, there was limited awareness of their scope and clinical application; that there is no legal duty to warn; and that the threat does not need to be imminent to warrant disclosure. No association between confidence and ‘good’ theoretical or applied clinical knowledge was identified. Uncertainty of their professional responsibility was identified and in the several case examples of active non-disclosure that were reported this uncertainty reflected the need for further understanding of the guidelines in regard to the processes required before disclosure was initiated.

**Conclusions:**

There is a need for further education and training about the guidelines associated with the legislation that would be relevant to support disclosure. The findings may inform future strategies to support introduction of policy changes in other jurisdictions where similar regulatory regimes are introduced.

## Background

Individuals receiving genetic test results are frequently the gatekeepers of information that might be relevant to their genetic relatives. Indeed, the dissemination of genetic information within a family may be impacted by numerous factors, such as divorce, family estrangement, adoption, and wanting to prevent stress and anxiety [1, 2]. However, some probands (that is, the first person in a family in whom the genetic nutation is identified) or another family member where there is incapacity in the proband, choose not to communicate the information regarding their test results and their potential impact to at-risk relatives; others fail to despite their intention to do so and having opportunities where this may have occurred [3]. Hereafter the former is referred to as active non-disclosure and the latter as passive non-disclosure. Accordingly, genetically at-risk relatives may be deprived of the chance to make informed choices about whether to seek genetic testing and take action in light of results [1, 4]. Previous research indicates that even when an individual recognises his or her responsibility to convey pertinent genetic information to relatives, the communication process is not always straightforward [1]. Factors include wishing to avoid causing anxiety and distress among family members, or a lack of confidence about how to deliver the information [1]. Studies also highlight that genetic health professionals (GHPs; i.e. clinical genetics specialists (CG), genetic counsellors (GC) and other medical specialists with genetics expertise) are rarely informed of a proband’s intention to actively not disclose genetic test results [1, 5]. Where it is identified, active non-disclosure can often be addressed during counselling. However, passive non-disclosure often remains undiscovered [5].

Genetic counselling is inherently familial [2, 6]. Depending on the genetic condition in question, various concerns may arise as to the nature of the disclosure, its timing, identifying the individuals at risk and arbitration of who should be informed [1]. GHPs need to be aware of complex family dynamics and reasons for non-disclosure, to be able to counsel the proband appropriately and promote accurate and effective disclosure of pertinent information within the family [2, 7]. While it has been shown that in most cases, probands are willing to share genetic information with first-degree relatives [8, 9], they do not seem to feel a responsibility to inform family members with whom they are not in touch or to relatives more distantly related than first-degree [2, 8]. When faced with a situation in which a client refuses or otherwise fails to inform their at-risk family members, GHPs face a conflict [8] between their responsibility to maintain client confidentiality and their desire to prevent harm and promote the interests of at-risk relatives. An additional factor in disclosure processes is relatives’ autonomy and purported right not to know [2, 10, 11].

The ethical obligation to prevent harm to at-risk relatives and promote the duty of care by GHPs is in conflict with Privacy laws and professional regulations that prohibits disclosure of information to a third party without the consent of the proband (duty of confidentiality). Australia is a federated nation made up of six states, and 10 territories (two of which have a limited right of self-government). The federal Privacy Act (1988) applies to all health professionals working in commonwealth bodies and private sector companies with an annual revenue over 3 million dollars. This would include GHPs working in certain private practice settings, but not those working in state public hospitals - where the majority are currently employed. To address the ethical aspects of the duty of care in regard to at-risk genetic relatives and the conflict with the duty of confidentiality to the probands, in 2006, amendments to section 95AA of the Australian federal Privacy Act 1988 were introduced, allowing for the disclosure of relevant genetic information to at-risk genetic relatives without the client’s consent, if there is ‘reasonable belief’ that disclosure is necessary to “lessen or prevent a serious threat to the life, health or safety” of the relatives. Importantly, while the amendment permitted overriding the duty of confidentiality and allowed the disclosure without consent, the amendment did not confer a duty to warn. The amendment also marked a change in the definition of the threat, which previously required it be imminent. The amended Privacy Act enabled the Privacy Commissioner to approve Guidelines developed by the National Health and Medical Research Council, published in 2009 (and amended in 2014) [12], outlining a framework that provides medical practitioners working in the private sector with nine central steps that must be met when choosing to disclose necessary information without the proband’s consent.

However, as the majority of Australian GHPs work in state-based public hospitals, they are not bound by the commonwealth Act. GHPs working in the public system in each State and the two self-governing Territories operate under relevant legislation and regulatory instruments, but in most Australian jurisdictions there remains a lack of harmonisation with the federal legal instruments governing genetic non-disclosure [13, 14]. New South Wales (NSW) is an exception to this general status quo. In 2012 NSW introduced the *Health Legislation Amendment Act 2012* to amend several distinct Health Acts, including the *Health Records and Information Privacy Act 2002*. This allowed the NSW Information and Privacy Commissioner to approve the NSW Health Privacy Guidelines [15] which effectively synchronised NSW and federal regulation of the disclosure of genetic information by medical practitioners without consent. So, medical practitioners with clinical genetics expertise working in the NSW Public System as well as those in private practice are now effectively governed by the same regime for responding to non-disclosure. See Table 3 for a summary of the Guidelines.

Awareness or understanding of privacy guidelines governing the practice of GHPs is an important component of professional practice, but data from previous international studies suggests that this is lacking [10]. These studies also suggest that in practice GHPs do not break client confidentiality as often as previously indicated in hypothetically-based studies [16–18], acknowledging that most non-disclosure cases are not intentional (passive) and often resolve on their own after counselling, time and assistance [19]. There are both legal and ethical aspects of disclosure without consent, described by Dove et al. (2019) as co-existing duties: “a duty of confidentiality owed to the patient and a putative duty of care to the patient’s close relatives” [20]. There is international debate over a GHPs’ legal duty to warn relative/s where protection of a proband’s privacy and confidentiality may lead to potential serious harm for a relative/s [13, 21]. Illustrative of this is the ongoing UK legal case of *ABC v St George NHS Trust* where a father who had recently been diagnosed with Huntington disease refused consent for his neurology team to disclose this genetic information to his pregnant daughter. Disclosure was subsequently and unintentionally made to ABC that her father had Huntington disease (and therefore she was at 50% risk of also developing the condition) after she had given birth [20]. The current appeal is ongoing. Initially the case that the Defendants (St George NHS Trust) had a relationship with the Claimant (ABC) leading to a duty of care: “to take reasonable steps to prevent the claimant from suffering injury” and that the Defendants should have provided information “in a timely manner when it was known, or ought to have been known, that the Claimant was pregnant” and that the Claimant should have been given the opportunity for “urgent diagnosis and testing” was struck out – see EWHC 1394 (QB) ([2015]) [22]. The Claimant (ABC) appealed to the Court of Appeal, who allowed the appeal against the striking out, deciding that she did have an arguable case in negligence (and under the Human Rights Act 1998- see [2017] PIQR P15, [2017] EWCA Civ 336 [23]. The matter was sent back to the High Court for trial which is currently in process. The findings have the potential to impact regulation in jurisdictions not only in the UK but more broadly as it will be important for GHPs to be clear of their roles, responsibilities and boundaries [14]. Thus, exploration of NSW GHPs’ awareness of, or whether they act in accordance with, guidelines introduced to facilitate disclosure of genetic information without consent may inform any future support needed with the introduction of such legislation in other jurisdictions. To this end, this study was designed to evaluate three central elements in relation to GHPs and the NSW Health Privacy Guidelines: (1) awareness, (2) knowledge of the guidelines’ content, limits and clinical application, and (3) confidence to take on the role of disclosure without consent.

## Methods

### Sample and recruitment

GHPs were eligible to participate if they had worked for over a year (or one-year full-time equivalent) in the role of a genetic counsellor, a clinical geneticist or other medical specialist with genetics expertise (such as in cancer or laboratory genetics) in NSW within the 3 years previous to the study implementation (June 2016). Participants were recruited via e-mails through Listservs to members of the NSW Genetic Counsellors’ Network (NGCN), the NSW branch of the Australasian Association of Clinical Geneticists (AACG) and the NSW branch of the Human Genetics Society of Australasia (HGSA). Additionally, the study was promoted during genetics services journal club meetings in two Sydney tertiary hospitals, and at two conferences in 2016: HGSA and Familial Aspects of Cancer: Research and Practice. Participants implied their consent through submitting their questionnaire after having acknowledged that they had read the Participant Information as approved by the University of Sydney’s Ethics Committee (see Additional file 1).

### Survey instrument

The online questionnaire was developed by the research team, piloted and hosted via Survey Monkey (Survey Monkey Inc. San Mateo, California, USA). Items were guided by the literature [10, 16–18]. The survey was divided into five different sections – demographics, awareness and theoretical knowledge, clinical scenarios to assess application knowledge, attitudes and confidence, and previous experience with clients who actively refused to notify at-risk relatives.

Scenarios.

Four representative clinical scenarios were included. These are summarised in Table 1, with full scenarios in Additional file 1, questions 11–14.

**Table 1:** Summary of case scenarios

Number	Case	Scenario summary
1	Genetic risk with implications to genetic relatives	A 26-year old woman affected with breast cancer and a *BRCA1* mutation carrier who actively refused to inform her older sister of the potential risk
2	Genetic risk with no implications to genetic relatives, but potential threats to relatives’ offspring	A woman identified as an X-linked haemophilia carrier, following the birth of an affected son, who actively refused to inform her sister of her potential carrier status and possibility of an affected pregnancy.
3	Genetic risk with implications to genetic relatives and relatives’ offspring	A woman identified as a Fragile X carrier, following the birth of an affected son, who actively refused to inform her sister of potential carrier status and risks, and possibility of an affected pregnancy.
4	Genetic risk with implications to genetic relatives and their unborn children	A 29-year old man identified with a balanced chromosome translocation, following an investigation of his partner’s history of recurrent miscarriages, who actively refused to inform his sister of her risk of having the same translocation and potentially multiple miscarriages.

### Embedded education

After submitting their questionnaires, participants were provided with a link to a questionnaire answer sheet (developed by the research team) relating to the survey items that tested knowledge (see Additional file 2) and a link to a Centre for Genetics Education webpage containing the *NSW Guidelines* Fact Sheet [24] .

### Data collection and analysis

Data collection was anonymous and took place over 4 months. Three reminders were sent out during this period: 1) 2 weeks after the survey opening, 2) 4 weeks after the survey opening, 3) 3 months after initiating data collection. The data was downloaded into Excel and SPSS v 23.0 (IBM Corp: SPSS Statistics for Windows Version 23.0, Arnouk, NY, IBM Corp) for analysis. After data cleaning, theoretical knowledge (questions 9–10) and clinical application knowledge (questions 11–14) data was dichotomised into correct (1) or incorrect/ unsure (0) responses. A cumulative knowledge score was calculated and scores above the median for non-parametric data or above the mean for parametric data were classified as “good knowledge” (1) or “poor knowledge” (0). Chi square tests were used to identify associations between demographics, confidence and knowledge scores.

## Results

### Sample

In total, 37/ 44 responses were valid for analysis. Of the 7 invalid responses, one was blank, and six others were rejected as they did not meet the inclusion criteria or omitted demographic data. Respondents included clinical geneticists/medical specialists with genetics expertise (*n* = 8) and 29 genetic counsellors (including a social worker working in a genetics service) (see Table 2). The majority reported being employed in a genetic counselling role, most had at least 2 years of experience in the field of clinical genetics, and nearly half had worked in the field for at least 11 years. The majority of the respondents were employed in roles in the public sector only: with approximately half working in specialty areas, a quarter in general genetics only, and a third were working in both general and specialty areas.

**Table 2:** Participants’ demographic information

Total number of participants	43
Profession	
HGSA Certified Genetic Counsellor	15
Associate Genetic Counsellor	14
Clinical Geneticist/Medical specialist with genetics expertise	8
Area	
General genetics only	9
Specialty only (e.g. cardiac, familial cancer, prenatal, etc.)	14
Both general and specialty	14
Years of practice in the field of genetics	
0–1 years	4
2–5 years	9
6–10 years	8
11–15 years	9
16+ years	7
Workplace	
Currently not practicing as a genetic health professional	1
Metropolitan genetics service	30
Regional genetics service	4
Outreach genetics service	
Sector	
Public only	31
Private only	3
Both public and private	3
Gender	
Female	29
Male	8

### Knowledge of guidelines

Two thirds of respondents (23/37) reported having read the NSW Health Privacy Guidelines prior to completing the survey, and many (22/37), demonstrated “good knowledge” of the Guidelines, scoring above the median of 9/14 (range 4–13) (Table 3). A significant association was identified between “good” theoretical knowledge score and having read the guidelines (χ^2^ = 8.91. df = 1, *p* < 0.05). No association was found between a ‘good’ theoretical knowledge score and any of the demographic factors.

**Table 3:** Australian Federal and NSW State guidelines on the use and disclosure of genetic information to a patient’s relatives for health professionals and theoretical and clinical application of the Guidelines

GUIDE LINE	DESCRIPTION	THEORETICAL KNOWLEDGE				APPLIED KNOWLEDGE			
		QN	CORRECT	INCORRECT	UNSURE	QN	CORRECT	INCORRECT	UNSURE
1	Use or disclosure of genetic information without consent may proceed only when the authorising medical practitioner has a reasonable belief that this is necessary to lessen or prevent a serious threat to the life, health or safety of a genetic relative.	10a	14	19	4	11A	24	9	4
		10d	10	15	2	11B	26	6	5
						13A	11	20	6
						13B	3	26	8
	New South Wales Health, 2014, p. 5	10c	17	6	14	12A	26	6	5
						12B	13	15	9
						14A	13	13	11
						14B	16	12	9
2	Specific ethical considerations must be taken into account when making a decision about whether or not to use or disclose genetic information without consent.	10e	34	0	3				
3	Reasonable steps must be taken to obtain the consent of the patient or his or her authorised representative to use or disclose genetic information.	10e							
4	The authorising medical practitioner should have a significant role in the care of the patient and sufficient knowledge of the patient’s condition and its genetic basis to take responsibility for decision-making about use or disclosure.	10b	28	14	6				
5	Prior to any decision concerning use or disclosure, the authorising medical practitioner must discuss the case with other health practitioners with appropriate expertise to assess fully the specific situation.	10b	24	2	11				
6	Where practicable, the identity of the patient should not be apparent or readily ascertainable in the course of inter-professional communication.	10j	31	1	5				
7	Disclosure to genetic relatives should be limited to genetic information that is necessary for communicating the increased risk and should avoid identifying the patient or conveying that there was no consent for the disclosure.	10i	36	0	1				
		10j	31	1	5				
		10k	9	9	19				
8	Disclosure of genetic information without consent should generally be limited to relatives no further removed than third degree relatives.	10f	25	4	8				
9	All stages of the process must be fully documented, including how the decision to use or disclose without consent was made.	10m	36	0	1				

All but one participant (36/37) were aware that all steps of the process, including reasoning to disclose information to genetic relatives, should be fully documented (guideline 9) and that disclosure without consent should be limited to information that is necessary for notifying a relative of an increased genetic risk (guideline 7). Most (31/37) were also aware that disclosure should avoid identifying the client if possible (guidelines 6 and 7) and 34/37 that specific ethical considerations (guideline 2) must be deliberated during the decision-making process. Many (25/37) were aware that disclosure without consent should generally be limited to recipients no further than third-degree relatives (guideline 8).

However, only half of participants (19/37) were aware that the proband should be notified of the disclosure unless there is ‘contradictory indication’ not to do so (guideline 8); and only 17/37 were aware that disclosure without consent does not apply to genetic information that solely presents a threat to an unborn child, but that it does apply if there is a psychosocial risk to a pregnant woman (New South Wales Health, 2014, p. 5). Only 19/37 participants were aware that the guidelines are relevant in the public and private sector. Imminence of a “threat to the life, health and safety” of a genetic relative is not a requirement for disclosure without consent (guideline 1), however, only 14/37 correctly recalled this. Further, only 10/37 participants were aware that while the guidelines facilitate disclosure without consent, there is no legal obligation to use or disclose a client’s genetic information to lessen or prevent a serious threat to genetic relatives (that is, no duty to warn and override the duty of confidentiality) (NSW Health, 2014, p. 5). Lastly, only 9/37 participants were certain that disclosure should avoid conveying that there was no consent for disclosure (guideline 7).

The majority of participants (28/37) were aware that the authorising health professional in the process of disclosure without consent must be a medical practitioner who assumes a significant role in the care of the client (guideline 4). In addition, 24/37 participants were aware that discussions with other health professionals with appropriate expertise are required during the process of deciding whether disclosure without consent is appropriate (guideline 5). However, only 17/37 were aware that the authorising medical practitioner is able to identify other appropriately trained professionals such as genetic counsellors to assume the role of disclosure (NSW Health, 2014, p. 25: Table 3).

### Clinical application of the guidelines

In comparison to theoretical knowledge, only 18/37 were considered to have “good” knowledge of the clinical application of the Guidelines, scoring above the mean of 3.77 + 1.6 (SD) for the eight questions related to the four clinical scenarios summarised in Table 1.

#### Scenario one (Q11A, 11B)

##### Genetic risk with implications to genetic relatives – identification of a BRCA1 mutation potentially presenting a threat to a current first-degree relative

Most participants (24/35) correctly identified that disclosure without consent of an increased genetic risk, would be in accordance with the NSW Privacy Legislation (2012) and Health Privacy Guidelines, as there are measures to lessen or prevent the threat to relatives (as there is a relevant family history). Most (26/35) also recognised that disclosing information without consent, solely on the grounds that there is potential harm to relatives’ future children, would not be in accordance with the Guidelines.

#### Scenario two (Q12A,12B)

##### Genetic risk for an X-linked condition with no implications to current genetic relatives but a potential threat to future genetic relatives

Where the genetic risk (i.e. X-linked haemophilia) presents no direct threat to a potential carrier, most participants (26/35) correctly identified that disclosure without consent would not be in accordance with the guidelines.

#### Scenarios three and four (Q13A, 13B and 14A, 14B)

##### Genetic risk for Fragile X or a balanced chromosome translocation with implications to current and/or future genetic relatives

If the genetic risk in question (i.e. Fragile X or balanced chromosome translocation), presents a threat to a current genetic relative such as primary ovarian insufficiency (due to Fragile X) and higher miscarriage rate (due to a balanced translocation), disclosure without consent *would* be in accordance with the NSW Guidelines as there are measures to lessen or prevent the threat to at-risk relatives. However, only 11/35 (Fragile X scenario) and 3/35 (balanced translocation scenario) participants correctly recognised that disclosure was appropriate. Only 13/35 (Fragile X) and 16/35 (balanced translocation) participants correctly identified that disclosure in the interest of relative’s future children would not be in accordance with the Guidelines. There was no association between ‘good’ theoretical and ‘good’ applied clinical knowledge, however there was an association between ‘good’ applied clinical knowledge score and years in practice (χ^2^ = 12.21, df = 4, *p* < 0.05). There was no association with any other demographic factor.

### Attitudes and confidence

Only a medical practitioner can authorise disclosure without consent. However, only 5/7 practicing clinical geneticists/medical specialists correctly identified that this was their role and two were unsure. In addition, only 6/27 genetic counsellors correctly identified that they are *not* authorised under the Guidelines to take on the lead role of disclosure (they can assume the role if authorised by a medical practitioner); 9/27 responses were incorrect, 14/27 participants were unsure. While 19/35 reported feeling confident in their knowledge to properly manage the process of non-disclosure without consent, there was no association between confidence and ‘good’ theoretical knowledge or ‘good’ applied clinical knowledge. However, 31/35 expressed interest in further education and training on managing the process of disclosure without consent.

### Experience with active non-disclosure

In total, 24/32 participants thought they would encounter 1–3 cases of active non-disclosure over the next 12-months. Thirteen participants (6 certified genetic counsellors; 3 associate genetic counsellors /social worker, and four clinical geneticists/medical specialists; 8 with > 10 years-experience), provided comments on a particular case or generic comments on examples of non-disclosure seen. Overall, participants highlighted four main points about active non-disclosure: (1) it does not occur frequently, (2) health professionals often have no way of knowing whether a client has disclosed genetic information to at-risk relatives, (3) health professionals will rarely have relatives’ contact information, and (4) most cases of active non-disclosure resolve with time and discussion, or via an affected relative. For example:

*“The above are the only two cases of active non-disclosure that I have encountered (that patients have admitted to me) in 20 years in genetics. In both instances, there was no way for me to contact the at-risk relative. This is by far the most likely scenario.”* (P2, CG).

Of the 7 case examples provided (Table 4), the most common examples of active non-disclosure involved parents who did not communicate their results to a minor, or adult children (*n* = 3). Reasons included to protect a minor, or the proband did not want to worry young adults. There were also probands reported (*n* = 4) who did not want to disclose their child’s or their own genetic status results to at risk relatives (including siblings and cousins). Reasons included: the proband did not want to disclose their child had a condition; it was seen as the proband’s own business; the proband felt their relative(s) would not share genetic information with them, or estrangement. Although 2/7 of these self-reported cases remained unresolved, and 1/7 was only resolved after a relative became pregnant, others resolved when other affected relatives were identified, or a genetics health professional supported the client in writing their own family letter.

*“… over the course of a few contacts with the patient, I challenged [X’s] beliefs and we worked together to write a letter that could be distributed to the family anonymously.”* (P10, GC).

**Table 4:** Examples of active non-disclosure reported by participants

ROLE	EXPERIENCE	CONDITION	PROBAND	REFUSED TO DISCLOSE TO	REASON FOR NON-DISCLOSURE	DISCLOSURE
Clinical Geneticist P42	16+ years	Carrier of a severe X-linked disease (female)	Parent	Daughter	Parent refused	RESOLVED – informed when pregnant.
Genetic counsellor (cert) P29	11–15 years	Hereditary Creutzfeldt Jacob Disease (Fhx) gene mutation carrier (female)	Parent	Children	Unable to disclose as did not want to cause concern	UNRESOLVED
Genetic counsellor (cert) P22	2–5 years	Lynch syndrome	Parent	Children	Worry, guilt, anxiety for children	UNRESOLVED via genetic counselling
Genetic counsellor (Associate) P25	6–10 years	Balanced translocation	Female	At-risk relatives	Did not want to disclose child had a genetic condition, felt family would treat child differently	RESOLVED via other relatives
Genetic counsellor (cert) P20	6–10 years	Myotonic dystrophy (DM1)	Adult male	Relatives	It was only his (proband’s) business	RESOLVED via other relatives
Genetic counsellor (cert) P12	6–10 years	Not-specified	Female	Cousins	They wouldn’t care about me	RESOLVED via ongoing genetic counselling
Genetic counsellor (Associate) P35	2–5 years	Cancer (male)	Male	Extended Family members	Did not have a close and open relationship with extended family	RESOLVED via ongoing genetic counselling -wrote a family letter

A number of participants gave an overview of reasons for active non-disclosure that had been reported to them. These included parents refusing to tell their children to protect them; treatment fatigue; dealing with a diagnosis/prognosis; being overwhelmed; and not understanding the result and implications for relatives of a cancer diagnosis. One example of passive non-disclosure was also provided where the proband wanted to wait until they themselves were ready to disclose.

### Views on the guidelines

A few participants provided feedback: 1). The guidelines do not consider the future harm to a parent of birth of a child with a genetic condition. 2). There is no guideline regarding disclosure to a non-biological relative who may nevertheless be a relevant individual, for example pregnant women. 3). Those who engage in non-disclosure are unlikely to provide contact details for relatives. 4). Maybe it is sufficient to know that the guidelines and legislation exist, and one can easily access that information as and when required.

*“…..There’s not much point in me memorising the details of legislation which may well have changed by the time I need to use it (if ever). It’s enough for me to know that rules exist and to have the intention that if the situation ever arises, I will find out what they are, and apply them.”* (P45, CG).

## Discussion

The majority of NSW GHPs who participated in the study demonstrated ‘good’ theoretical knowledge of content of the NSW Health Privacy legislation and Guidelines [15]. This was associated with having previously read the guidelines. However, ‘good’ knowledge of how the guidelines apply clinically was only associated with the number of years in practice. Nevertheless, results suggest that many GHPs lacked understanding of the scope and clinical application of guidelines regarding disclosure of genetic information without consent in NSW, especially in circumstances of a less direct threat, or disclosing genetic risks presenting threats to an unborn child. Therefore, it is not surprising that half of the participants did not feel confident in their knowledge to manage the process of disclosure without consent and were uncertain of their professional responsibility.

Research conducted by Clarke et al. (2005) suggest that active non-disclosure is uncommon [1]. Communication barriers within a family are commonly addressed during consultations, where healthcare professionals support probands to promote adequate dissemination of genetic information. While participants in the study reported here described previous experiences with potentially active non-disclosure, they indicated that many instances are able to be resolved with discussion and time, although a few cases remained unresolved, even after follow-up. Several clinical geneticists/medical specialists were unsure if it was their role to take on professional responsibility for disclosing without consent, and a third of genetic counsellors incorrectly believed they had a professional responsibility and they had the appropriate authority. Further clarification and education about this process would therefore be beneficial.

Traditionally, GHPs have been trained to follow an individual rather than a familial approach to disclosure, in which they provide clients with information and support that allow each individual to make their own independent choices [2, 6]. When faced with a client who actively refuses to disclose information to at-risk relatives, it has been reported that professionals are more likely to prioritise perceived client autonomy over the responsibility to family members [10]. Strategies recommended by participants in this study included use of a communication tool to identify cases of active or passive non-disclosure that would benefit follow up. Other online resources to facilitate communication have been recommended and supported by governing legislation in some countries such as France [19, 25, 26].

### Practice implications

Technological advances, expanding knowledge and mainstreaming in genetics are likely to raise more ethical and professional challenges for all health professionals. It is therefore vital that health professionals continue to carefully consider proband’s interests but also recognise that in genetics there may also be responsibilities to relatives too – depending on circumstances. The increasing need to balance the dual duties to maintain the confidentiality of the patient and limit the harms to at-risk genetic relatives brings in to focus both legal and ethical issues and cases such *ABC* vs *St George* may inform health professionals’ ongoing legal responsibilities [13, 20, 22, 23]. Therefore, the data presented in this study should be regarded as a first step in identifying potential areas of importance to highlight for GHPs (or other health professionals engaging in the provision of genetic information) regarding current privacy law and guidelines informing clinical practice, and how best to promote future changes so as to increase health professionals’ theoretical and practical knowledge. The study also highlights the level of complexity regarding this issue and the need for further clarification around legal obligations and permissions of healthcare professionals (guidelines 1, 4, 5), when there is a threat to an unborn child(ren) (guideline 7) and disclosure process (guideline 8). Healthcare professionals could benefit from further education and support to facilitate clinical application of guidelines when dealing with non-disclosure in practice. Given the differences in Federal and State \Territory legislation and guidelines in Australia, an Australia-wide approach is likely to be more effective; with training through national websites, webinars, conferences, seminars, and more interactive sessions [14].

### Research recommendations

Primary healthcare professionals and medical specialists are the gatekeepers between clients and genetic services. As genetics is progressively implemented into clinical practice (including non-genetics specialities), it is likely that a wider range of health professionals will be ordering genetic tests and providing genetic counselling. Future studies should explore the experiences and skills of non-genetic health professionals in dealing with potential conflicts between client confidentiality and responsibilities to genetic relatives. It will be extremely important to involve these professionals in future research, as educational tools and comprehensive guidelines, stating their responsibilities and limitations, will need to be targeted to their specific needs and practice.

### Study limitations

While it is estimated that there are 153 practicing genetic counsellors/clinical geneticists/medical specialists listed as NGCN, AACG and/or HGSA members working in NSW, it is difficult to determine the exact response rate. We were unable to monitor the number who received an e-mail invitation for the survey or were aware of the survey through promotion at the conferences, and how many opted not to participate.

## Conclusions

There are ongoing ethical and legal debates as to whether healthcare professionals have a responsibility to genetic relatives to disclose genetic information when there is a serious risk. When a proband fails to disseminate genetic information to at-risk relatives, healthcare professionals must balance the client’s interests (i.e. privacy and autonomy) against genetic relatives’ putative right to know. In this small Australian study, many professionals were aware and understood various aspects of the relevant legal Guidelines, however, there was uncertainty of the scope and clinical application of the current legislative and regulatory framework. The Australian health system is a mix of both private practice and government (public) services engendering complexity in the regulatory framework which may have added to the uncertainty and which may not be present in countries with a health service that is largely public. Nevertheless, these findings suggest that if legislation for disclosure to genetic relatives without consent is introduced, there is a need for education to increase awareness and support genetic health professionals in the application of the underpinning guidelines within their healthcare system. Additionally, effective education and training strategies are likely to be relevant for introduction of further legislation and guidelines in the future, especially around interpretation of nuanced exceptions and professional responsibility for implementation of recommendations in the clinic.

## Supplementary information

**Additional file 1.** Survey Instrument.

**Additional file 2.** Answer Sheet.

## Data Availability

All de-identifed interview transcripts are digitally stored and stored by the University of Sydney’s archive system. Professor Barlow-Stewart takes responsibility for the data to be made available.

## Abbreviations

AACG: NSW branch of the Australasian Association of Clinical Geneticists
CG: Clinical genetics specialists
GC: Genetic counsellors
GHPs: Genetic health professionals
HGSA: NSW branch of the Human Genetics Society of Australasia
NGCN: NSW Genetic Counsellors’ Network
NSW: New South Wales
