# Supplementary material for: Disclosure to genetic relatives without consent – Australian genetic professionals’ awareness of the health privacy law
Source: BMC Med Ethics. 2020 Feb 4;21:13. doi: 10.1186/s12910-020-0451-1 (PMC7001268; doi:10.1186/s12910-020-0451-1)
Supplement: Supplementary file 1 — Additional file 1. Survey Instrument. [file 12910_2020_451_MOESM1_ESM.docx]

**Disclosure to genetic relatives without consent – Australian genetic professionals’ awareness of the health privacy law**

**Additional Information File 1: Survey Instrument**

Participant Information

1. I have read and understood the information provided in the Participant Information Statement, which is attached with the invitation letter. I currently or have practiced in NSW within the last 3 years.


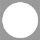
 Yes

**Section I Demographics**

2. Profession:


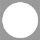
 HGSA Certified Genetic Counsellor


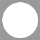
 Associate Genetic Counsellor


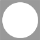
 Clinical Geneticist/Medical specialist with genetics expertise


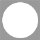
 Student


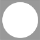
 Other (please specify)

3. Area:


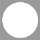
 General genetics only


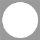
 Specialty only (e.g. cardiac, familial cancer, prenatal, etc.)


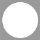
 Both general and specialty

4. Years of practice in the field of genetics:


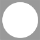
 0-1 year


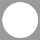
 2-5 years


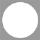
 6-10 years


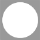
 11-15 years


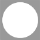
 16+ years

5. Which scenario best describes your workplace?


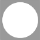
 Currently not practicing as a genetic health professional


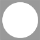
 Metropolitan genetics service


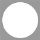
 Regional genetics service


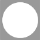
 Outreach genetics service


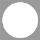
 Other (please specify)

6. What type of sector do you work in?


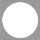
 Public only


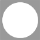
 Private only


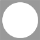
 Both public and private


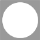
 Other (please specify)

7. Gender


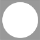
 F


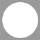
 M


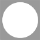
 X


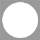
 Prefer not to say

**Section II Awareness and Knowledge**

8. Prior to this survey have you read the *NSW Information & Privacy Commission Guideline: Use &* *disclosure of genetic information without consent*, published in October 2014?


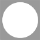
 Yes


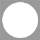
 No


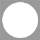
 Unsure

9. The *NSW Information & Privacy Commission Guideline: Use & disclosure of genetic information without consent* supports the NSW Health Legislation Amendment Act 2012, which applies to the practice of health professionals working in which of the following sectors?


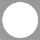
 Public Sector only


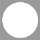
 Private Sector only


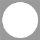
 Public and Private Sectors


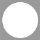
 Unsure

10. In regard to the privacy amendments in the *NSW Health Legislation Amendment Act 2012*, please answer true, false or unsure to the following statements:

| a. Disclosure without consent can only occur when there is a serious threat to life, health and safety, which is imminent. | True | False | Unsure |
| --- | --- | --- | --- |
| b. The authorising health professional must be a medical practitioner who has had a significant role in the care of the patient. |  |  |  |
| c. Disclosure without consent applies to situations concerning genetic information that presents a serious threat to an unborn child. |  |  |  |
| d. There is a legal obligation to use or disclose a patient's genetic information without consent where there is a 'reasonable belief' that such disclosure is necessary to lessen or prevent a serious threat to the life, health or safety of an individual. |  |  |  |
| e. Ethical considerations must be taken into account prior to deciding whether it is appropriate to disclose relevant genetic information. |  |  |  |
| f. The recipient of the information must be a genetic relative of the individual generally no further than third-degree relatives. |  |  |  |
| g. The authorising medical practitioner may choose to identify another suitable professional to take on the role of disclosure. |  |  |  |
| h. Prior to disclosure the authorising medical practitioner is not required to discuss the case with other health practitioners with appropriate expertise, if he/she is certain of the decision. |  |  |  |
| i. Disclosure to genetic relatives should be limited to information that is necessary for communicating an increased genetic risk. |  |  |  |
| j. Disclosure to genetic relatives should avoid identifying the patient. |  |  |  |
| k. Disclosure to genetic relatives should convey that there was no consent for disclosure. |  |  |  |
| l. The patient should be notified of the disclosure unless there is 'contradictory indication' not to do so. |  |  |  |
| m. All steps of process and reasoning should be entirely documented. |  |  |  |

**Section III Scenarios**

The next set of questions relate to the scenarios provided. In this section we wish to explore your views on disclosure of relevant genetic information.

11. Anna is a 26-year old woman who was recently diagnosed with breast cancer and had treatment focused genetic testing. A BRCA1 mutation was identified. Anna has a sister, Rebecca, who is two years older. Anna has not told Rebecca that she has a BRCA1 mutation and that Rebecca is also at risk of having it. Over some time you have tried to ensure a reasonable effort has been made to encourage Anna to tell Rebecca about the identified mutation and Rebecca's potential risk, or assist in her doing so, but Anna refuses to notify her sister. You have Rebecca's contact details. The following actions are in keeping with the amendments to the NSW Privacy Legislation (2012):

1. Disclose relevant genetic information to Rebecca, without Anna's consent, as there are measures to lessen or prevent the threat to Rebecca.


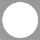
 Yes
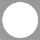
 No
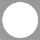
 Unsure

1. Disclose relevant genetic information to Rebecca, without Anna's consent, as there is potential harm to Rebecca's future children.


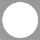
 Yes
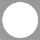
 No
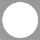
 Unsure

12. Julie has been identified as an X-linked carrier for haemophilia following the birth of her affected son. She has a sister, Rachael, who is of reproductive age. However Julie has not told Rachael that her nephew has haemophilia or that she is also at risk of being a carrier. Over some time you have tried to ensure a reasonable effort has been made to encourage Julie to tell Rachael about her identified carrier status and Rachael’s own potential risk, or assist Julie in doing so, but she refuses to notify her sister. You have Rachael’s contact details. The following actions are in keeping with the amendments to the NSW Privacy

Legislation (2012):

1. Disclose relevant genetic information to Rachael, without Julie’s consent, as there are measures to lessen or prevent the threat to Rachael.


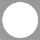
 Yes
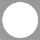
 No
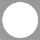
 Unsure

1. Disclose relevant genetic information to Rachael, without Julie’s consent, as there is potential harm to Rachael’s future children.


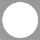
 Yes
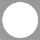
 No
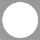
 Unsure

13. In the scenario above, if Julie’s son was diagnosed with Fragile X syndrome instead of haemophilia, and Julie has been identified as an X-linked carrier, which of the following actions are in keeping with the amendments to the NSW Privacy Legislation (2012):

1. Disclose relevant genetic information to Rachael, without Julie’s consent, as there are measures to lessen or prevent the threat to Rachael.


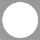
 Yes
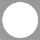
 No
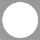
 Unsure

1. Disclose relevant genetic information to Rachael, without Julie’s consent, as there is potential harm to Rachael’s future children.


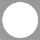
 Yes
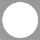
 No
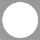
 Unsure

14. George, a 29 year-old man was found to have a balanced chromosome translocation during evaluation for his partner’s history of recurrent miscarriages. The translocation diagnosed was considered to be unlikely to cause him any medical problems but may have accounted for his partner’s history of miscarriages. George stated that there was no family history of children with major congenital malformations or disabilities. George’s sister Eleni is at risk of having the same translocation and potentially multiple miscarriages because of her carrier status. George was provided with this information. He refused to advise Eleni although she had recently had a miscarriage and was known to be planning another pregnancy. The following actions are in keeping with the amendments to the NSW Privacy Legislation (2012):

1. Disclose relevant genetic information to Eleni, without George’s consent, as there are measures to lessen or prevent the threat to Eleni.


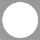
 Yes
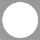
 No
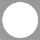
 Unsure

1. Disclose relevant genetic information to Eleni, without George’s consent, as there is potential harm to Eleni’s future children.


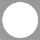
 Yes
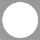
 No
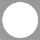
 Unsure

**Section IV Attitudes**

15. To what extent do you agree with the following statements:

| I feel confident that I have enough knowledge to work with my team to appropriately manage the process of disclosure without consent (where appropriate). | Strongly disagree | Disagree | Unsure | Agree | Strongly agree |
| --- | --- | --- | --- | --- | --- |
| It is my professional responsibility to provide disclosure without consent where appropriate and I feel authorised to do so. |  |  |  |  |  |

16. I would like further education and training on managing the process of disclosure without consent.


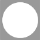
 Yes


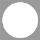
 No


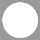
 Unsure

**Section V Experience**

17. In practice, non-disclosure is often passive. How often do you predict that you would encounter a patient who would actively refuse to inform at-risk relatives (over the next 12-months)?


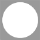
 Never


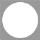
 1


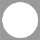
 2


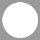
 3


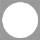
 4+

18. This question aims to assess the skills and knowledge you might have used in a situation of active refusal.

In the text box below, if applicable, please describe an event in a non-identifying way where a patient actively refused to notify at-risk relatives. You may like to include information about the patient’s reason for refusing to disclose, actions or strategies taken by you and how the situation was resolved.
