# Supplementary material for: Disclosure to genetic relatives without consent – Australian genetic professionals’ awareness of the health privacy law
Source: BMC Med Ethics. 2020 Feb 4;21:13. doi: 10.1186/s12910-020-0451-1 (PMC7001268; doi:10.1186/s12910-020-0451-1)
Supplement: Supplementary file 2 — Additional file 2. Answer Sheet. [file 12910_2020_451_MOESM2_ESM.docx]

**Disclosure to genetic relatives without consent – Australian genetic professionals’ awareness of the health privacy law**

**Additional Information File 2: Answer Sheet**

Frequently Asked Questions (FAQ): NSW Health Privacy Guidelines

To whom do the NSW Health Privacy Guidelines and the NSW Health Legislation Amendment Act 2012 apply?

They apply to health professionals working in the Public Sector.

When is it acceptable to disclose genetic information without consent?

Use or disclosure of genetic information without consent may proceed only when the authorising medical practitioner has a reasonable belief that this is necessary to lessen or prevent a serious threat to the life, health or safety of a genetic relative.

What should be considered prior to disclosure without consent?

Specific ethical considerations must be taken into account when making a decision about whether or not to use or disclose genetic information without consent.

What steps should be taken prior to disclosure without consent?

Reasonable steps must be taken to obtain the consent of the patient or his or her authorised representative to use or disclose genetic information.

Who can take responsibility for decision-making about disclosure without consent?

The authorising medical practitioner should have a significant role in the care of the patient and sufficient knowledge of the patient’s condition and its genetic basis to take responsibility for decision-making about use or disclosure.

Who should be consulted in the decision-making process about disclosure without consent?

Prior to any decision concerning use or disclosure, the authorising medical practitioner must discuss the case with other health practitioners with appropriate expertise to assess fully the specific situation.

Should the identity of the patient be disclosed?

Where applicable, the identity of the patient should not be apparent or readily ascertainable in the course of inter-professional communication.

What should be disclosed?

Disclosure to genetic relatives should be limited to genetic information that is necessary for communicating the increased risk and should avoid identifying the patient or conveying that there was no consent for disclosure.

Who should the information be disclosed to?

Disclosure of genetic information without consent should generally be limited to a relative no further removed than third-degree relatives.

What should be documented?

All stages of the process must be fully documented, including how the decision to use or disclose without consent was made.

<https://www.genetics.edu.au/health-professionals/FAQNSWHealthprivacyGuidelines.pdf/view>
